# Insights into medical students’ perceptions of work culture during the COVID-19 pandemic: a mixed method study

**DOI:** 10.1186/s12909-023-04936-4

**Published:** 2024-01-03

**Authors:** Stephane Mouchabac, Vladimir Adrien, Thomas Diot, Marie-Christine Renaud, Alain Carrié, Alexis Bourla, Florian Ferreri

**Affiliations:** 1 Department of Psychiatry, Saint-Antoine Hospital, Sorbonne University, AP-HP, Paris, F-75012 France; 2 Infrastructure for Clinical Research in Neurosciences (iCRIN), Paris Brain Institute, Paris, France; 3 Faculty of Medicine, Sorbonne University, Paris, France

**Keywords:** Work culture, Medical students, COVID-19, Professional identity, Coping

## Abstract

**Background:**

The COVID-19 pandemic brought about profound social changes that affected students worldwide. These changes had both psychological and economic consequences, and also led to the adoption of new teaching methods. It can also have an impact on work culture, which is the collective set of values, norms, and practices within a specific profession, shaping how individuals in that field behave, communicate, and identify with their work. The aim of the study was to examine medical students’ perception of professional culture during the COVID-19 crisis when they voluntarily participated in the healthcare network established, outside of university placements, for the management of COVID patients.

**Methods:**

A questionnaire study based on the vignette methodology was conducted among third-year medical students. Drawing from three scenarios in which students were variably engaged in crisis management, it included questions about their perceptions of the medical profession, their motivation, and their sense of belonging to the profession.

**Results:**

352 students responded to the survey. The pandemic had both a positive and a negative impact on students’ perceptions of the medical profession. Cluster analysis using a *k*-means algorithm and principal component analysis revealed three clusters of students with different perceptions of the medical profession. The first cluster, which represented the majority of students, corresponded to a relatively positive perception of the profession that was reinforced during the pandemic. In the second cluster, students’ perceptions were reinforced still further, and particular importance was attached to field experience. Students in the third cluster had the most negative perceptions, having been shaken the most by the pandemic, and they attached little importance to field experience.

**Conclusions:**

The analysis highlighted the importance of students being able to adapt and draw on a range of resources during the COVID-19 pandemic. This underscores the need for work cultures that support adaptability and coping. Further research is needed to understand its long-term effects on students’ perceptions of the medical profession and to identify interventions that could support students in the aftermath of this difficult period.

**Supplementary Information:**

The online version contains supplementary material available at 10.1186/s12909-023-04936-4.

## Background

The COVID-19 pandemic was a major event for people around the world. It brought about very profound social changes, because of both its economic impact and the various measures that were taken to manage the health crisis (quarantine, lockdown, curfew, closure of schools, shops and businesses). Students were particularly affected by it, in terms of its psychological consequences [1, 2] and economic impact [3], as well as the resulting changes in teaching methods. It also raised questions about career building and, by extension, *work culture*.

Based on concepts borrowed from the sociology of work, the notion of *work culture* is most often associated with a specific economic activity. It is assumed that the exercise of a professional activity sustainably and deeply influences individuals and contributes to their development [4]. For students, entering the *practical* phase of their course and engaging in more specialized training can be regarded as a form of *secondary socialization*, in that they internalize the functioning of new universes. At this stage, they are already able to define themselves partly in terms of belonging to a profession, that is, a fully-fledged social group which therefore has its own culture [5]. A work culture is essentially a culture that is passed down from generation to generation. However, it includes the notion of companionship, not least in medical education [6, 7]. A work culture can be learned either formally, through courses or internships that transmit knowledge and *know-how*, or by mimicking how exemplars speak and act [8–11]. These aspects of work culture facilitate relationships between those who possess them. They reinforce the feeling of belonging to the same group because those who have gone through this process construct the meaning of their experiences in a similar way and do not need to explain everything. The practice of a professional activity involves mastering a set of tools and techniques (processes), corresponding to distinctive skills shared by the group. It is therefore a way of positioning oneself in relation to other professionals. Finally, members of a profession have specific representations of their work culture, such as how it should be implemented, as well as its purpose and its place in the work organization. This type of professional socialization requires a specific language that is adapted to the profession’s technical requirements and allows its members to express their representations of it. A work culture is capable of identically coding certain experiences so that they then become common, and of eliciting specific behaviors.

These considerations are particularly valid in the medical profession, which has a culture with a strong identity component (corporation, order, specialty, competition-type rites of passage) [12–14] and whose skills are shared and tacitly recognized (knowledge of physical examination, investigative techniques, clinical reasoning, etc.) and which only they are authorized to perform. In theoretical pedagogy, students can acquire specific knowledge; however, the most profound impact on their professional perspectives and behaviors is likely to arise from socialization processes [15]. Regarding language, all physicians know how to speak to patients, and how to communicate about patients with their colleagues (e.g., nurses and social workers [16]).

In France, medical studies are organized in three cycles. The first cycle lasts 3 years: a common core curriculum in the first year, followed by a 2-year diploma course in medical sciences. In the third year, students begin their daily hospital or outpatient internships and participate in the clinical and administrative activity of the care units. The second cycle (diploma in advanced medical sciences) also lasts for 3 years. In the third cycle, which lasts 4–5 years, students train in their specialties (residency).

To minimize the impact of the pandemic on medical students, urgent changes had to be made to the organization of their education. In most countries, there was a rapid transition to online teaching, virtual simulations, and technology-enhanced coaching for healthcare providers [17], or else a structural reorganization [18]. However, little is known about the pandemic’s impact on students’ perceptions of the work culture.

In many countries, medical students were mobilized to support the healthcare system at the height of the pandemic. However, a survey of 4870 Indonesian medical students found that only 48.7% of respondents were willing to volunteer, and only 18.7% felt ready to practice, as they did not think they had sufficient knowledge and skills [19]. Furthermore, volunteering may influence the perception of professional roles and identification with the profession [20], so entering practice [21] may accelerate the construction of a professional identity [22]. A mixed-methods study among 900 Indian students showed that despite the COVID-19 pandemic, the majority of respondents (63.4%) retained a positive image of the profession, and over 91.4% of them confirmed their career choice [23].

Nevertheless, another study showed that students who engaged in an elective COVID-19 literature review course while their faculty was closed continued their literature searches after the return of face-to-face classes [24].

In France, students could perform various tasks on a voluntary basis, such as providing back-up in intensive care units or tracing patients under the COVISAN system, where one of the objectives was to support people who were screened and their contacts, both to ensure their care and to contain the disease. Other students were trained in nursing skills and could be assigned to departments under particular strain. Finally, students were confronted with end-of-life support and the fear of having to make ethical choices at the peak of the pandemic.

These experiences potentially constituted motivational reinforcers that influenced students’ acquisition of the medical profession’s work culture. A better understanding of the mechanisms that can influence this acquisition either positively or negatively could help to improve current teaching methods. Recent studies have mainly focused on the negative impact of the pandemic from a psychological point of view [25], and not on aspects that may have promoted a form of resilience.

To explore these concepts, we adopted the mixed qualitative-quantitative *vignettes* method, which has long been used in the social, behavioral and health sciences. The objective of the present study was to measure the effect of students’ engagement in the healthcare system during the pandemic, and to explore whether it reinforced their professional identity.

## Methods

In normal times, second- and third-year medical students in France have a choice of 27 optional courses, but the COVID-19 lockdown led to a reorganization of teaching, and at Paris-Sorbonne University, the medical faculty committee replaced all these options with a single optional course.

As part of this course, students were asked to think about work culture in the context of COVID-19. There was no prior theoretical teaching on this subject before the questionnaire; so the students were “naive” on this subject so as not to influence the answers.

We conducted a two-part cross-sectional questionnaire study. The first part consisted of the analysis of three vignettes featuring short descriptions of real-life situations where medical students were involved in COVID-19 crisis management. These vignettes were provided on the inter-university remote assessment system (SIDES®) platform, and students had to react to them spontaneously, without any preparation. This first part was not scored.

In the second part, students were provided with educational documents in French (10–15 pages). In order to work on a more personal and theoretical reflection, they then had to produce a written summary (1–2 pages) on the “Impact of the COVID-19 pandemic on work culture”, using the concepts defined in the documents. The results of part 1 have not been given to avoid influencing personal reflection.

### Vignette development

The classic vignette method has two components: (a) development and use of vignettes; and (b) collection of information on participants’ more specific characteristics, to use as covariates in the analysis of the vignette data [26, 27].

In general (and in our study), vignettes are short, hypothetical scenarios. They are used as a stimulus to provoke reaction and discussion. They make it possible to elicit and analyze participants’ beliefs, attitudes, and judgments in a different way from a classic questionnaire, the argument being that they occur within a context, rather than in isolation [28]. We believed that this technique was relevant to the focus of our study, having already used it in other areas [29, 30].

Based on the literature, and with the help of a sociology laboratory expert in this field, we identified the dimensions of the medical profession work culture dimensions (Table 1) [26].

**Table 1:** Dimensions of the medical work culture

Dimension	Subdimension
Representations (R)	Perception of the realities of the profession (R1) Reference to the whole (= organization), that is, all aspects of the job (R2) Being in one’s place / implicitly supporting the organization (R3)
Way of thinking (W)	Membership (W1) Image of work: its purposes and how the work should be done (W2) Impact on personal construction (W3)
Skills (S)	Development of distinctive and inclusive skills (vs. others) (S1) Natural skills (S2)
Language (L)	Technical language (L1) Socially distinctive (allowing for identification with *outside*) (L2) Acquisition through mimicry / lessons / knowledge transfer (L3)
Technical dimension (T)	Specific tools (utensils/language) (T1) Techniques/Specific processes (knowledge) (T2)
Values (V)	Sense of belonging (V1) Control against intrusion by third parties: we only understand *if we are here* (V2)

We then developed three vignettes covering these different dimensions, working with sociologists to ensure the relevance of the data we had collected and developing a sociological hypothesis. The questions were elaborated with the help of two focus groups including psychiatrists and sociologists, and tested with cross-validation (one psychiatrist and one sociologist from the team who had not participated in the construction of the vignette and the questions) to ensure that they each corresponded to one dimension of the work culture [27], if the desired dimension was not found, the item was reworked in the group. The aim was to probe the various dimensions of the work culture and the way in which they interacted with students’ involvement in COVID-19 crisis management. The use of three vignettes allowed us to test these dimensions from different perspectives. It also increased the validity of the tool.

Each of our vignettes described a specific situation: volunteering to work for a COVISAN support platform (Vignette 1); impossibility of supporting teams on the front line owing to personal health issues (Vignette 2); and potential need to make ethical choices about patient care (Vignette 3). The themes addressed in each vignette and the items explored in the questions are set out in Table 2.

Each vignette was followed by 10 questions designed to address the dimensions of work culture in relation to the vignette. Participants responded on a 5-point Likert scale ranging from *Totally disagree* to *Totally agree*.

**Table 2:** Subdimensions measured in each of the three vignettes

Vignette topics	Subdimension measured
Volunteering to work for a COVISAN support platform - Following theoretical and practical training, and after immersion, the student may join a mobile team - Important for the student’s commitment as a future physician, providing an opportunity to explore other dimensions of the medical profession - Negative reactions of the student’s nonphysician relatives (worried about the risk of the student catching COVID-19 and advising against participation)	- R1, R2, R3 - W3 - S1, S2 - V1, V2 - L1 - T2
- Following accelerated training in nursing skills - Impossibility of reinforcing care teams owing to relatives’ medical problems (e.g., father at risk of severe form of COVID-19) and student’s own pathology - Student fears being stigmatized because of inaction - Loss of special financial bonus	- R1 - W1, W2 - S1 - T2 - L2 - V1, V2
- Potentially having to make ethical choices over patient care in ICU - Student learns that an intensive care society of a foreign country is preparing to make decisions about patient selection to favor those with a greater chance of survival - Ethical conflict, as the student has been left unprepared by culture and training for reasoning on the basis of triage criteria - Representations about the COVID-19 pandemic (more social than medical; situation not comparable to an earthquake or bombing).	- R1, R2, R3 - W2, W3 - L4 - S1 - V2

### Participants

A total of 369 third-year students enrolled on the optional course, and 352 completed both parts of the study. Respondents were informed that their responses in Part 1 would form the basis for the summary in Part 2. We couldn’t know (anonymity of replies) whether they had taken part in the various voluntary activities themselves.

### Data collection

The study was conducted under the approval from Medecine-Sorbonne University (APHP_SU) institutional academic review board (N1_05/05/20) in accordance with the University guidelines and regulations for research. As anonymity was essential for students to respond freely, no student identifiers were recorded. Students provided their age and gender at the start of the questionnaire.

Because of the lockdown, the study was conducted on line, via the SIDES® platform. This platform is shared by all French medical schools, allowing each of them to conduct/administer their exams via digital tablets or computers completely autonomously. It should be noted that SIDES® complies with the General Data Protection Regulation EU 2016/679 and the 1978 French Data Protection Act. All participants on SIDES® were informed that the data could be processed anonymously for research purposes.

After a short introductory text, each vignette was presented to participants, followed by a set of questions. For Part 1, a session was opened on SIDES® over a long time window extending from 8 a.m. on 25 May 2021 to 11 p.m. on 29 May 2021. Students had 30 min to respond. Responses were retrieved in the form of an Excel file.

### Statistical analysis

Descriptive analyses were performed with R (version 4.2.2). We calculated the mean scores for each of the six dimensions, across all three vignettes, and then for each vignette. To identify student profiles that shared common responses across different dimensions measured by the questionnaire, we employed a clustering method. These algorithms leverage the richness of the initial data to attempt to divide individuals into a predetermined number of groups, where individuals within each group share common characteristics. We used the *k*-means algorithm to identify groups of students according to their work culture perceptions. This algorithm started by randomly selecting *k* students to define the center of the *k* cluster. The distance of each observation from the center was calculated within each cluster, and observations were placed in the cluster where they were closest to the center. Each time a cluster was modified, the center was recalculated and the distance was measured between every observation and the center. If necessary, observations were moved from one cluster to another. This process was repeated until convergence was reached. The algorithm was rerun several times, as the initial choice of cluster center can affect the final result [31]. After all the reruns, the resulting clusters were compared and we retained the best distribution. The final number of clusters was chosen within regard to statistical tools [32], and the coherence of the results was addressed by authors.

We compared item scores and dimensions between clusters to assess the items on which the clusters differed the most. We ran two-tailed tests with a significance level of *p* < 0.05. Each cluster was assigned a label based on its score on each dimension, as well as on significantly different items. Descriptive statistics with age and gender were calculated to compare the clusters.

## Results

### Overall analysis

**Fig. 1:**
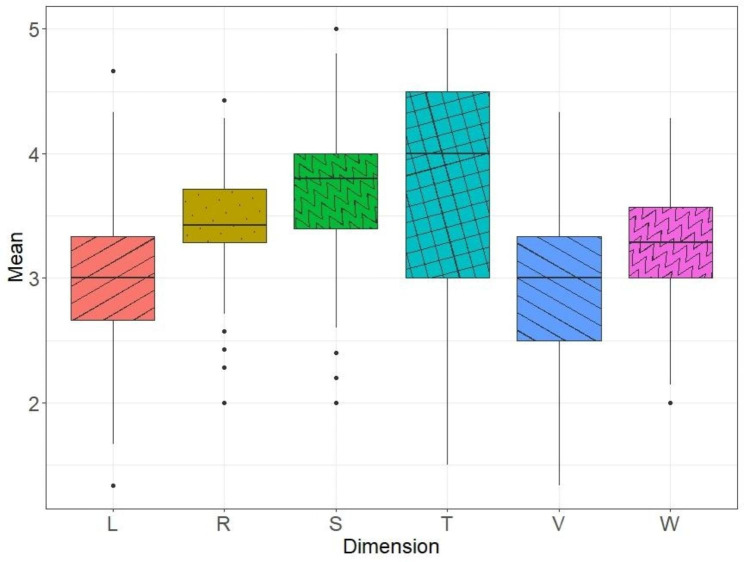
Boxplot of Dimension Scores Across All Three Vignettes. The box represents the interquartile range, with a horizontal line drawn in the middle to indicate the median. The whiskers represent the highest and lowest value excluding outliers, while dots beyond the extreme line shows potential outliers. L: Language; R: Representation; S: Skills; T: Technical dimensions; V: Values; W: Way of thinking

All students participated in the study (369 in the promotion), but full data was available for 352 students, representing 95% of the third-year medical students. The data from the 17 students who failed to complete the three vignettes were excluded from analyses. They did not differ significantly from the included students on either gender (42% vs. 64.8% female) or mean age (24.7 years, *SD* = 0.77 vs. 24.2 years, *SD* = 2.6). The mean scores for each dimension are set out in Fig. 1. The most dispersed scores on the Likert scale were on the technical and skills dimensions. Scores on the language and values dimensions were more centrally distributed, while the other four dimensions mostly had high scores. Despite this, the dimension scores showed significant heterogeneity, with high standard deviations indicating wide ranging responses from students.

### Contextual importance of vignette

**Fig. 2:**
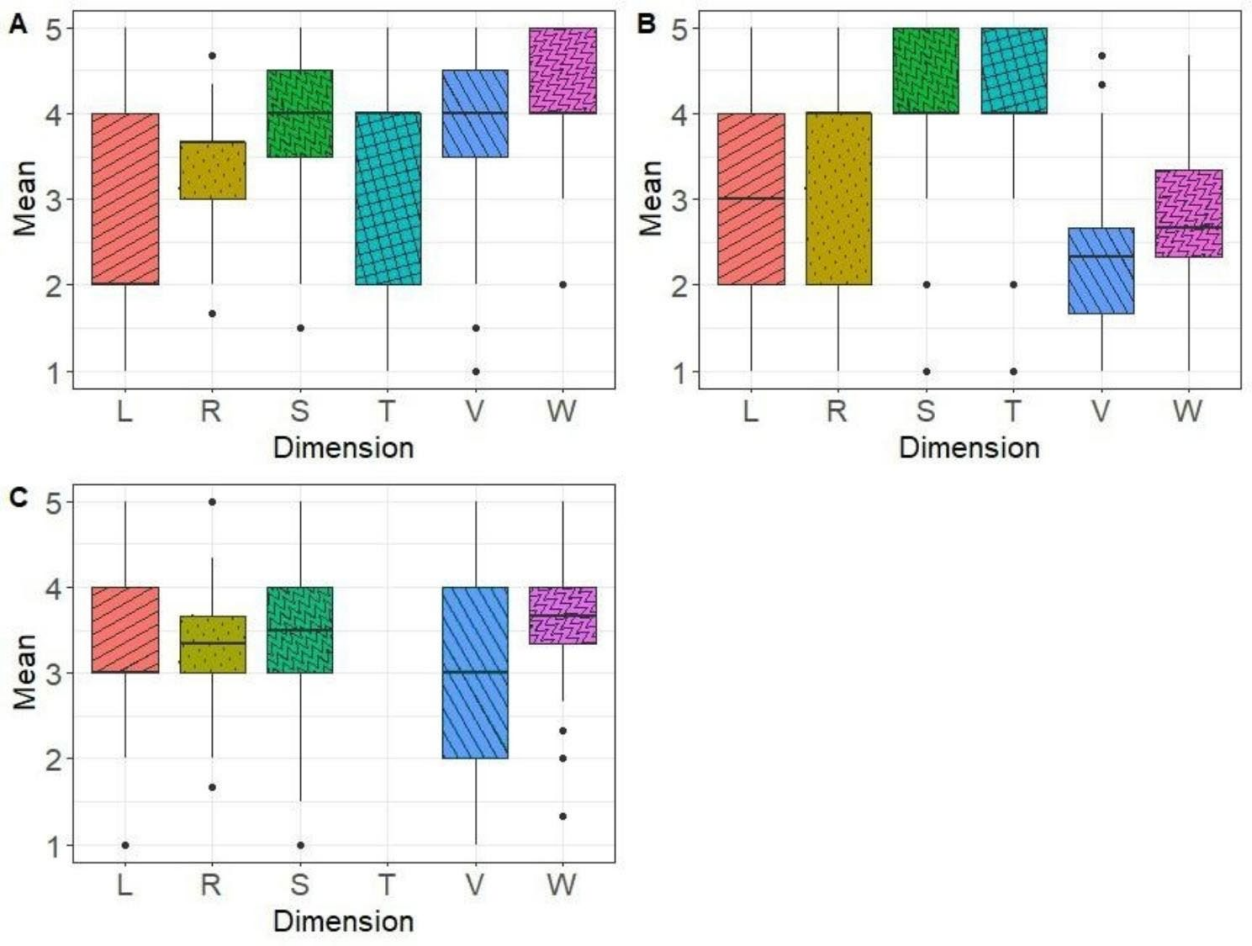
Dimension Scores for Each Vignette (**A**: Vignette 1; **B**: Vignette 2; **C**: Vignette 3)

Mean dimension scores for each vignette are shown in Fig. 2. We found significant differences in the values of each dimension depending on the vignette.

The box represents the interquartile range, with a horizontal line drawn in the middle to indicate the median. The whiskers represent the highest and lowest value excluding outliers, while dots beyond the extreme line shows potential outliers. L: Language; R: Representation; S: Skills; T: Technical dimensions; V: Values; W: Way of thinking.

We ran a confirmatory factor analysis on all the responses from all the vignettes, to study the relevance of the six dimensions of work culture. It was performed with the principal component analysis algorithm. This analysis did not reveal the dimensions we used to create the vignettes. Based on the previous figure, and in order to understand this result, we ran a separate factor analysis for each vignette. This revealed the initial dimensions, but also significant associations between several dimensions within the individual vignettes (results not presented). In the first and second vignette analyses, the skills and technical dimensions were associated. In the second and third vignette analyses, the values and way of thinking dimensions were associated. Finally, in the first and third vignette analyses, the representations and language dimensions were associated.

### Clustering

Cluster analysis using the *k*-means algorithm revealed three distinct clusters of students. We used several statistical indicators to determine the optimum number of clusters, including the total within sum of squares, gap statistic, and silhouette method. These indicators suggested that either three or four clusters would be appropriate. The final number of clusters was determined according to the size of each cluster and the relevance of the results to the research question.

**Fig. 3:**
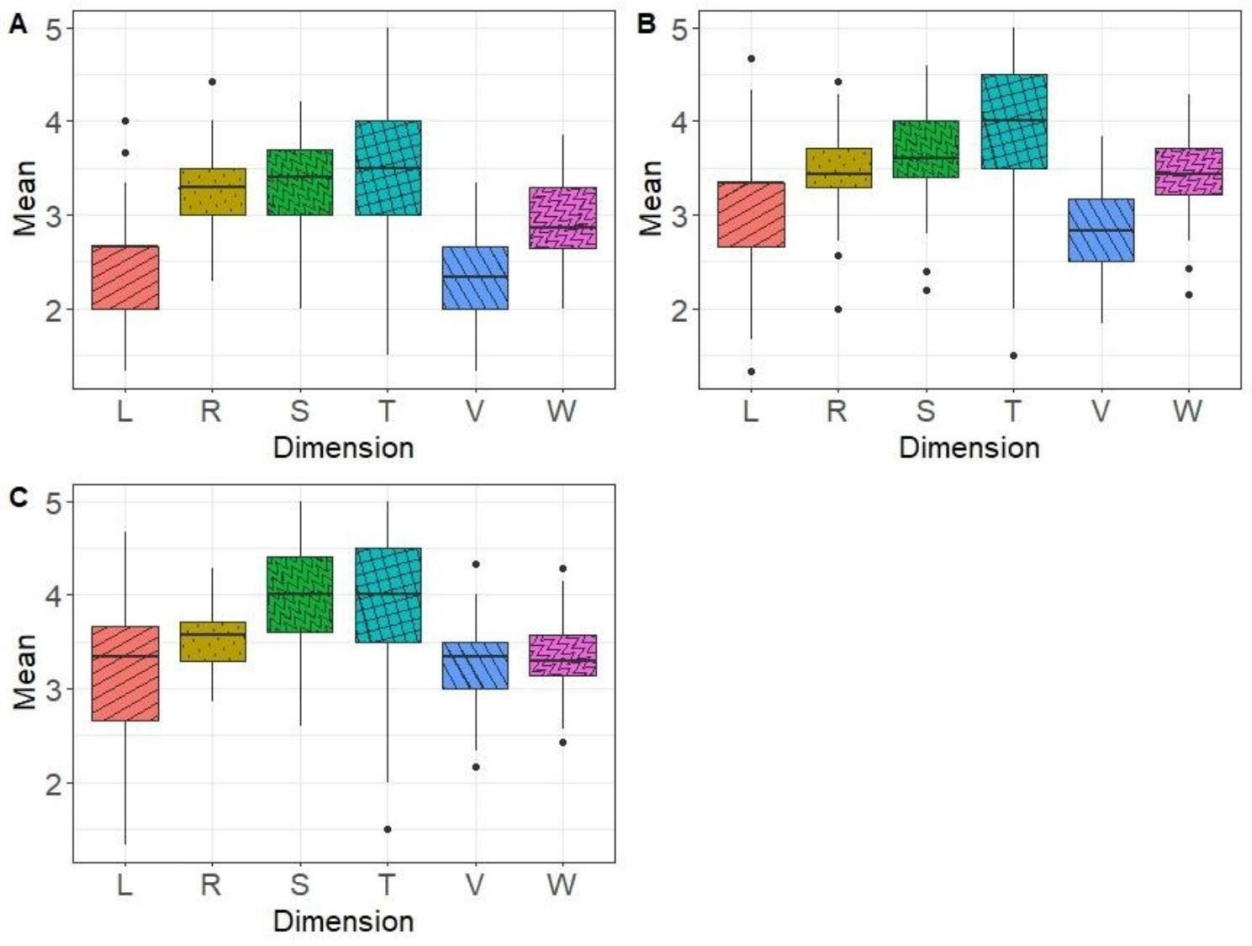
Dimension scores per cluster (**A**: Cluster 1; **B**: Cluster 2; **C**: Cluster 3)

The mean dimension scores for the three clusters we identified are provided in Fig. 3. Statistical tests on dimension scores both across and within the three vignettes revealed significant differences between the clusters on both dimensions and sociodemographic variables (age, gender) (see Table 3).

The box represents the interquartile range, with a horizontal line drawn in the middle to indicate the median. The whiskers represent the highest and lowest value excluding outliers, while dots beyond the extreme line shows potential outliers. L: Language; R: Representation; S: Skills; T: Technical dimensions; V: Values; W: Way of thinking.

**Table 3:** Mean Dimension Scores and Sociodemographic Characteristics According to Cluster

	C1 (*n* = 134)	C2 (*n* = 90)	C3 (*n* = 128)	*p*
**Dimension**				
Language (L)	2.47	3.13	3.18	< 0.05
Representations (R)	3.24	3.49	3.55	< 0.05
Skills (S)	3.32	3.7	3.97	< 0.05
Technical dimension (T)	3.53	3.87	3.77	< 0.05
Values (V)	2.33	2.83	3.27	< 0.05
Way of thinking (W)	2.95	3.46	3.36	< 0.05
**Demographic**				
Female gender	97 (72)	36 (40)	90 (70)	< 0.05
Age	24.1 (2.6)	24.5 (2.9)	24.2 (2.4)	< 0.05

**Fig. 4:**
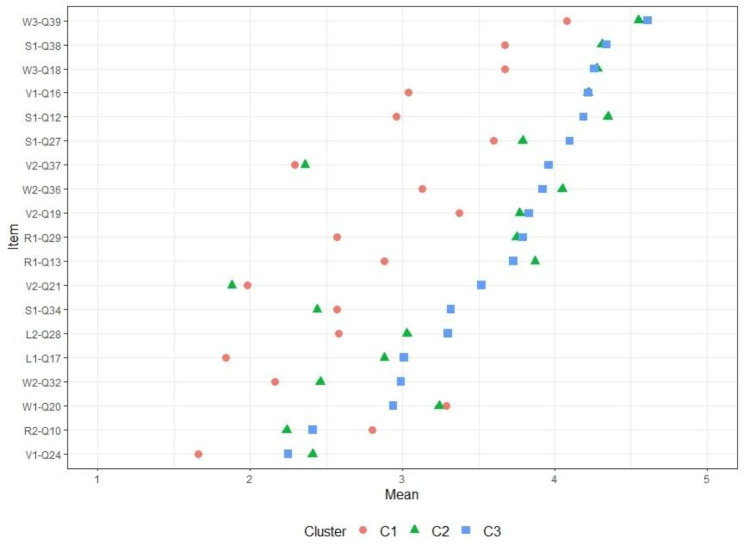
Significant Differences Between Clusters on Mean Item Scores

To probe differences within each dimension in each cluster, we compared the three clusters on each item. The most significantly different items are listed in Fig. 4. Centered around Cluster 3, this graph shows the patterns of students’ responses within each cluster. All item comparisons are available in the Additional files.

Students in Cluster 1 (*n* = 134, 38%) were labeled *reasonable*. They differed significantly on the skills dimension, as well as on several items in the representation dimension. These students agreed more with the idea that participating in the crisis workforce allowed them to acquire new skills, both social and technical. They also agreed more with the idea that participation allowed them to acquire an overview of the medical profession. Their perceptions of the pandemic differed from those of the third cluster, as they stated that the pandemic would have a lasting impact on the practice of medicine. Contrary to participants in Cluster 3, but like those in Cluster 2, they believed that enrolment potentially modified the feeling of belonging to a group, and even led to stigmatization (i.e., if people could not participate because of a disability, they were still stigmatized). Not being involved for whatever reason resulted in exclusion. Like those in Cluster 3, they were mostly female, with a mean age of 24.1 years.

Cluster 2 students (*n* = 83, 26%) were labeled *learners*. The idea that the experience equipped them with new skills was more pronounced in this cluster than in Cluster 1. Unlike those in the other two groups, they believed that the field experience allowed them to form an objective opinion. They also agreed strongly with the idea that only people who participated in these activities could understand practical difficulties such as having to make ethical choices. However, they differed from the first cluster on the issue of taking risks in participating in medical activities, as they agreed less with the idea that a student with health issues (either own medical condition or vulnerable relatives) should be recruited at the same time as the others if the need is not immediate. This cluster differed significantly from the two others on demographics, as it was mostly made up of male students (60%, *p* < 0.05).

Clusters 1 and 2 mostly had the same patterns of answers, but with different intensities for each item and each dimension. They greatly contrasted with students in Cluster 3 (*n* = 142, 36%), who could be labeled *cautious* or *conservative*. Regarding participation, they agreed less with the idea that it allowed students to develop a more effective language, gain an overview of their future profession, and acquire new skills through new practices, new jobs, or new relationships. Furthermore, they did not think that nonparticipation deprived students of knowledge they would otherwise have acquired. Nor did they think that this field experience allowed them to develop an objective opinion on the situations they encountered. On the contrary, they believed that participating in this side activity potentially deprived hospital departments of effective students. Regarding membership, they agreed less with the idea that participation strengthened students’ affiliation to the medical profession. For them, participation did not alter group cohesiveness, and they did not believe that students who participated risked being stigmatized and subsequently encountering integration difficulties. Regarding self-construction and representations of the nursing profession, they agreed less than the others that participating potentially changed perceptions. In other words, these students tended to think that field experience does not bring any new skills, and does not really change the practice of medicine, self-representations, or representations of the profession. They seemed to think that skills (e.g., ability to judge guidelines or not) are acquired through academic teaching, not experience.

Other clustering methods (e.g., hierarchical clustering with Ward’s method) were also applied, but did not yield any significant results.

## Discussion

The initial analysis of results yielded by the classic vignette method [26] revealed a high degree of heterogeneity in the dimension scores. This was unforeseen, as we had expected to observe greater consistency in the student’s answers. We therefore applied the *k*-means clustering method to further explore the data. This method yielded robust and noteworthy results, grouping the students into three distinct clusters, such that there were homogenous response profiles within each cluster and marked differences between the clusters.

These results revealed that students belonged to three distinct clusters, each with its unique characteristics and perspectives on the work culture of the medical profession. Participants in Cluster 1 (38% of the sample) could be characterized as having *reasonable* views. They differed significantly from Cluster 3 on the skills dimension, as they agreed more with the statement that participating in the crisis workforce allowed them to acquire new skills, both social and technical. They also agreed more that participation allowed them to gain an overview of the medical profession. Cluster 3 students (36% of the sample) could be characterized as *cautious* or *conservative*. Students in Cluster 2 (26% of the sample) could be labeled as *learners*, owing to their belief that participating in the crisis workforce allowed them to acquire new skills and develop objective opinions. However, they differed from Cluster 1 on the issue of taking risks in participating in medical activities, agreeing less that students with personal medical risk factors should have been recruited for the crisis workforce. Demographic data revealed a significant difference between the three clusters, as the learners were mostly men, probably owing to the patriarchal view of Western societies [33] that field experience should be undertaken by men without disabilities [34]. Another explanation would be that females show better affective empathy (driving prosocial behavior) and men show more utilitarian behavior although no gender differences appear for cognitive empathy [35].

One of the present study’s strongpoints is that it was conducted among young medical students who had had very little clinical experience prior to the pandemic. We had expected these voluntary practical experiences to strengthen group cohesiveness and reinforce students’ commitment to pursuing a career in medicine. The culture of the medical profession has been extensively studied among more experienced students and interns, and has typically yielded fairly consistent results in terms of perceptions of the profession and the value of hands-on experience [36–39]. In our study, however, one of the clusters (Cluster 3) was quite distinct from the others. The pandemic is known to have had a severe impact on students’ mental health, leading to high levels of anxiety and depressive symptoms [1, 2, 18, 25]. The fact that medical work on the field in times of pandemic is anxiety-provoking may have led students of Cluster 3 to be less eager to have their first medical experience.

One possible avenue for further research is the feasibility or otherwise of implementing specific support mechanisms, such as debriefing sessions, especially for students like the participants in Cluster 3 who reject the value of clinical experience almost entirely. This could help them understand and process their pandemic experiences better. We suggest that the identified clusters can serve as a foundation for tailoring teaching or supervision to these profiles by taking them into consideration.

The confirmatory factor analysis we conducted yielded surprising results that ran counter to our expectations. We had based the vignettes on the dimensions of medical work culture we had identified in the literature. The main advantage of this research method is that it allows the vignettes to be adjusted according to the context in which the study is conducted [27, 40]. The fact that the initial dimensions did not emerge when we considered all three vignettes together indicates that students’ reactions depended on the situation. The COVID-19 pandemic created unprecedented situations that upended medical students’ perceptions. Faced with difficult and novel situations, such as the need for rapid ethical decision making, a divide emerged between medical students, particularly those with the profiles of participants in Clusters 1 and 3.

The factor analysis also revealed that some vignettes elicited responses involving multiple dimensions. We had expected these dimensions to be more isolated, given the way we formulated the questions. This underscores the importance of students being able to adapt to the new situations they encountered during the COVID-19 pandemic (coping) [41, 42] by drawing on a range of resources and aspects of the work culture.

Participation in the crisis workforce was voluntary and independent of the university. Although we do not know how many students in each cluster actually joined the crisis workforce, there are two interesting hypotheses regarding the COVISAN participation rate for the third cluster. The first is that students in this cluster participated less, and were therefore less aware of the benefits of these additional activities. This opened up a difference between the students, and we can postulate that the differing participation rates can explain differences in the students’ perceptions of the medical profession. The second hypothesis is that students in this cluster participated just as much as the other two groups, if not more so, but they had a negative experience. Participation may therefore have had a major impact on the perceived work culture of the medical profession, with these students being liable to value experience less in the future. Whichever hypothesis is correct, the COVID-19 pandemic may have had a negative impact and may not have reinforced the work culture for everyone.

The present study had several limitations. First, owing to the students’ anonymity, there was a lack of information about the characteristics of participants in each cluster, such as gender, age, and participation in the crisis workforce. This information would have been useful for identifying potential target groups for support measures based on the results of this study. Second, this study was conducted among young students, but it would have been interesting to explore the effects of the pandemic on older students’ perceptions of the medical profession, as well as those of other healthcare professionals. Although there have been several studies of this population, investigating the perceptions of different age groups and healthcare professionals would provide a more comprehensive understanding of the pandemic’s impact on the medical profession.

Overall, the findings of this study indicate that students held a range of perspectives on participation in the crisis workforce, with some seeing it as an opportunity to develop their skills and others viewing it with caution. Further research is needed to better understand the factors that contributed to these different perspectives.

### Electronic supplementary material

Below is the link to the electronic supplementary material.

Supplementary Material 1

Supplementary Material 2

Supplementary Material 3

Supplementary Material 4

## Data Availability

The vignettes and questions as well as the datasets used and analyzed during the current study are available from the corresponding author on reasonable request.

## References

[1] Moayed MS, Vahedian-Azimi A, Mirmomeni G, Rahimi-Bashar F, Goharimoghadam K, Pourhoseingholi MA (2021). Coronavirus (COVID-19)-Associated Psychological Distress among Medical students in Iran. Adv Exp Med Biol.

[2] Yu Y, She R, Luo S, Xin M, Li L, Wang S (2021). Factors influencing Depression and Mental Distress related to COVID-19 among University students in China: online cross-sectional mediation study. JMIR Ment Health.

[3] Tsurugano S, Nishikitani M, Inoue M, Yano E (2021). Impact of the COVID-19 pandemic on working students: results from the Labour Force Survey and the student lifestyle survey. J Occup Health.

[4] Culture de métier. et intégration post fusion-acquisition | Cairn.info. https://www.cairn.info/revue-gerer-et-comprendre1-2008-4-page-55.htm. Accessed 15 Nov 2022.

[5] Vieno K, Kuurne (née Ketokivi) (2022). Developing the Concept of belonging work for Social Research. Sociology.

[6] Huang K-Y, Chengalur-Smith I, Ran W. Not just for support: companionship activities in Healthcare virtual support communities. Commun Association Inform Syst. 2014;34.

[7] Li HO-Y, Bailey AMJ (2020). Medical Education amid the COVID-19 pandemic: New perspectives for the future. Acad Med.

[8] Leblanc S, Bouchot H, Secheppet M. Modélisation théorique de l’expérience mimétique et cours d’action: analyse de situations de formation en enseignement, santé, et sport. Activités. 2021. 10.4000/activites.6249.

[9] Barsalou LW (2008). Grounded Cognition. Annu Rev Psychol.

[10] Gallese V (2009). The two sides of mimesis: Girard’s mimetic theory, embodied simulation and social identification. J Conscious Stud.

[11] Billett S, Billett S, Harteis C, Gruber H (2014). Mimetic learning at work: learning through and across Professional Working lives. International Handbook of Research in Professional and practice-based Learning.

[12] Hafferty FW, Franks R (1994). The hidden curriculum, ethics teaching, and the structure of medical education. Acad Med.

[13] Thomas JC (2014). Re-visioning Medicine. J Med Humanit.

[14] Watling CJ, Ajjawi R, Bearman M (2020). Approaching culture in medical education: three perspectives. Med Educ.

[15] Shanafelt TD, Schein E, Minor LB, Trockel M, Schein P, Kirch D. Healing the Professional Culture of Medicine. Mayo Clinic Proceedings. 2019;94:1556–66. 10.1016/j.mayocp.2019.03.026 31303431

[16] Bourla A, Ferreri F, Ogorzelec L, Guinchard C, Mouchabac S (2018). [Assessment of mood disorders by passive data gathering: the concept of digital phenotype versus psychiatrist’s professional culture]. Encephale.

[17] Thakur A, Soklaridis S, Crawford A, Mulsant B, Sockalingam S (2021). Using Rapid Design thinking to Overcome COVID-19 challenges in Medical Education. Acad Med.

[18] Bazan D, Nowicki M, Rzymski P (2021). Medical students as the volunteer workforce during the COVID-19 pandemic: Polish experience. Int J Disaster Risk Reduct.

[19] Lazarus G, Findyartini A, Putera AM, Gamalliel N, Nugraha D, Adli I (2021). Willingness to volunteer and readiness to practice of undergraduate medical students during the COVID-19 pandemic: a cross-sectional survey in Indonesia. BMC Med Educ.

[20] Cerbin-Koczorowska M, Przymuszała P, Kłos M, Bazan D, Żebryk P, Uruski P (2022). Potential of volunteering in formal and Informal Medical Education—A theory-driven cross-sectional study with example of the COVID-19 pandemic. IJERPH.

[21] Gonnering RS (2010). Complexity theory and the puzzling competencies: systems-based practice and practice-based learning explored. J Surg Educ.

[22] Cruess RL, Cruess SR, Steinert Y (2016). Amending Miller’s pyramid to include professional identity formation. Acad Med.

[23] Vaz M, Ravindra SS, Chandran R, Ramachandra S, Timms S. O. The Covid-19 effect on medical students’ perceptions of their profession: a mixed methods study from South India. IJME. 2022;:01–12. 10.20529/IJME.2022.070 36420606

[24] Boscamp JR, Duffy CP, Barsky C, Stanton BF (2021). Medical students on the virtual Front line: a Literature Review Elective to provide COVID-19 clinical teams with essential information. Acad Med.

[25] Halperin SJ, Henderson MN, Prenner S, Grauer JN (2021). Prevalence of anxiety and depression among Medical Students during the Covid-19 pandemic: a cross-sectional study. J Med Educ Curric Dev.

[26] Jackson M, Harrison P, Swinburn B, Lawrence M (2015). Using a qualitative vignette to explore a Complex Public Health Issue. Qual Health Res.

[27] Atzmüller C, Steiner P. Experimental Vignette Studies in Survey Research. Methodology: European Journal of Research Methods for The Behavioral and Social Sciences. 2010;6:128–38.

[28] Grønhøj A, Bech-Larsen T (2010). Using vignettes to study family consumption processes. Psychol Mark.

[29] Bourla A, Mouchabac S, Ogorzelec L, Guinchard C, Ferreri F (2020). In collaboration with. Are student nurses ready for new technologies in mental health? Mixed-methods study. Nurse Educ Today.

[30] Bourla A, Ferreri F, Ogorzelec L, Peretti C-S, Guinchard C, Mouchabac S (2018). Psychiatrists’ attitudes toward disruptive New technologies: mixed-methods study. JMIR Ment Health.

[31] Celebi ME, Kingravi HA, Vela PA (2013). A comparative study of efficient initialization methods for the k-means clustering algorithm. Expert Syst Appl.

[32] Sugar CA, James GM (2003). Finding the number of clusters in a dataset. J Am Stat Assoc.

[33] Carter H (1994). Confronting patriarchal attitudes in the fight for professional recognition*. J Adv Nurs.

[34] Jain NR (2023). Legibility: knowing disability in medical education inclusion. Adv in Health Sci Educ.

[35] Christov-Moore L, Simpson EA, Coudé G, Grigaityte K, Iacoboni M, Ferrari PF (2014). Empathy: gender effects in brain and behavior. Neurosci Biobehavioral Reviews.

[36] Wilson I, Cowin LS, Johnson M, Young H (2013). Professional Identity in Medical students: Pedagogical challenges to Medical Education. Teach Learn Med.

[37] Monrouxe LV, Bullock A, Tseng H-M, Wells SE (2017). Association of professional identity, gender, team understanding, anxiety and workplace learning alignment with burnout in junior doctors: a longitudinal cohort study. BMJ Open.

[38] de Lasson L, Just E, Stegeager N, Malling B (2016). Professional identity formation in the transition from medical school to working life: a qualitative study of group-coaching courses for junior doctors. BMC Med Educ.

[39] Kalet A, Buckvar-Keltz L, Monson V, Harnik V, Hubbard S, Crowe R (2018). Professional identity formation in medical school: one measure reflects changes during pre-clerkship training. MedEdPublish.

[40] Silva A, Campos-Silva W, Gouvea M, Farina M (2019). Vignettes: a data collection technique to handle the differential operation of items in surveys. BBR.

[41] Roslan NS, Yusoff MSB, Morgan K, Ab Razak A, Ahmad Shauki NI (2022). What are the common themes of Physician Resilience? A Meta-synthesis of qualitative studies. Int J Environ Res Public Health.

[42] Sastrawan S, Newton JM, Malik G (2019). Nurses’ integrity and coping strategies: an integrative review. J Clin Nurs.

