# Supplementary material for: Insights into medical students’ perceptions of work culture during the COVID-19 pandemic: a mixed method study
Source: BMC Med Educ. 2024 Jan 3;24:21. doi: 10.1186/s12909-023-04936-4 (PMC10765811; doi:10.1186/s12909-023-04936-4)
Supplement: Supplementary file 1 — Supplementary Material 1 [file 12909_2023_4936_MOESM1_ESM.docx]

**Additional file 1 (PDF file extension): item means for each cluster.**

| **Dimension** | **Item** | **C1 (*n* = 134)** | **C2 (*n* = 90)** | **C3 (*n* = 128)** | ***p*** |
| --- | --- | --- | --- | --- | --- |
| Language | Q17-L1 | 2.88 | 1.84 | 3.01 | **< 0.05** |
|  | Q28-L2 | 3.03 | 2.58 | 3.3 | **< 0.05** |
|  | Q33-L3 | 3.49 | 2.98 | 3.24 | 0.16 |
| Representations | Q13-R1 | 3.87 | 2.88 | 3.73 | **< 0.05** |
|  | Q29-R1 | 3.75 | 2.57 | 3.79 | **< 0.05** |
|  | Q31-R1 | 2.94 | 3.27 | 3.18 | 0.82 |
|  | Q10-R2 | 2.24 | 2.8 | 2.41 | **< 0.05** |
|  | Q30-R2 | 4.01 | 3.61 | 3.85 | 0.18 |
|  | Q11-R3 | 4.35 | 4.29 | 4.51 | 0.06 |
|  | Q35-R3 | 3.27 | 3.27 | 3.39 | 0.32 |
| Skills | Q12-S1 | 4.35 | 2.96 | 4.19 | **< 0.05** |
|  | Q27-S1 | 3.79 | 3.6 | 4.1 | **< 0.05** |
|  | Q34-S1 | 2.44 | 2.57 | 3.32 | **< 0.05** |
|  | Q38-S1 | 4.31 | 3.67 | 4.34 | **< 0.05** |
|  | Q15-S2 | 3.61 | 3.8 | 3.92 | 0.23 |
| Technical dimension | Q14-T2 | 3.35 | 3.04 | 3.32 | 0.1 |
|  | Q26-T2 | 4.39 | 4.02 | 4.23 | 0.23 |
| Values | Q16-V1 | 4.22 | 3.04 | 4.22 | **< 0.05** |
|  | Q24-V1 | 2.41 | 1.66 | 2.25 | **< 0.05** |
|  | Q19-V2 | 3.77 | 3.37 | 3.83 | **< 0.05** |
|  | Q21-V2 | 1.88 | 1.98 | 3.52 | **< 0.05** |
|  | Q22-V2 | 2.35 | 1.64 | 1.87 | 0.48 |
|  | Q37-V2 | 2.36 | 2.29 | 3.96 | **< 0.05** |
| Way of thinking | Q20-W1 | 3.24 | 3.29 | 2.94 | **< 0.05** |
|  | Q23-W1 | 2.09 | 1.65 | 1.73 | 0.99 |
|  | Q25-W2 | 3.54 | 2.67 | 3.06 | 0.17 |
|  | Q32-W2 | 2.46 | 2.16 | 2.99 | **< 0.05** |
|  | Q36-W2 | 4.05 | 3.13 | 3.92 | **< 0.05** |
|  | Q18-W3 | 4.28 | 3.67 | 4.26 | **< 0.05** |
|  | Q39-W3 | 4.55 | 4.08 | 4.61 | **< 0.05** |

**Supplementary Table 1.** Item means for each cluster
