# Supplementary material for: Insights into medical students’ perceptions of work culture during the COVID-19 pandemic: a mixed method study
Source: BMC Med Educ. 2024 Jan 3;24:21. doi: 10.1186/s12909-023-04936-4 (PMC10765811; doi:10.1186/s12909-023-04936-4)
Supplement: Supplementary file 2 — Supplementary Material 2 [file 12909_2023_4936_MOESM2_ESM.docx]

Samy, a student in his 4th year, is doing an internship in hepatology. With the coronavirus crisis, the activity of the service has been totally reorganized. The closure of the beds in the ward where he works makes it possible for him to be assigned to another position, but he can also remain confined to his home (which would allow him to catch up on his courses). The faculty and the hospital then propose that the students join the COVISAN platform, whose objective is to provide effective support to patients with COVID19 at home. This is a voluntary and innovative approach that prefigures a new strategy to deal with the pandemic. The objective is to break the chains of transmission by identifying and isolating potentially contaminated people whose condition does not require hospitalization. Doctors think that this experience will have an impact on the students' personal development. Samy knows that he will have to follow a theoretical and practical training and after immersion, he may join a mobile team. He is both attracted by this option, which he thinks is important for his commitment as a future doctor and which will allow him to discover other dimensions of his profession, but he fears that he will not be up to the task. His relatives, who are not doctors, are worried that he will contract the coronavirus and advise him against participating.

1-Do you think that Samy's participation deprives a hospital service of a student who could have been useful to patients? (R2)

Strongly disagree

Somewhat agree

Neither agree nor disagree

Somewhat agree

Strongly agree

2- In your opinion, the involvement in COVISAN should be based only on voluntary work ?(R3)

Strongly disagree

Somewhat agree

Neither agree nor disagree

Somewhat agree

Strongly agree

3- Do you think that participating in COVISAN will provide Samy with different skills than the students who stayed in their internships? (S1 )

Strongly disagree

Agree a little

Neither agree nor disagree

Somewhat agree

Strongly agree

4-Do you think that COVISAN allows you to acquire a more global perception of the physician's job? (R1)

Strongly disagree

Agree a little

Neither agree nor disagree

Somewhat agree

Strongly agree

5-In your opinion, participating in COVISAN requires different knowledge than in the hospital ?(T2)

Strongly disagree

Somewhat agree

Neither agree nor disagree

Somewhat agree

Strongly agree

6- Do you think that as a 4th year student, Samy has the skills to go to patients' homes? (S2)

Strongly disagree

Agree a little

Neither agree nor disagree

Somewhat agree

Strongly agree

7-Do you think Samy's experience with COVISAN will strengthen his sense of belonging to the medical community? (V1)

Strongly disagree

Disagree a little

Neither agree nor disagree

Somewhat agree

Strongly agree

8-Do you think that after this mission, Samy will have acquired a more precise and efficient medical language? (L1)

Strongly disagree

Agree a little

Neither agree nor disagree

Somewhat agree

Strongly agree

9-Do you think that COVISAN can have an impact on Samy's (W3) personal development?

Strongly disagree

Agree a little

Neither agree nor disagree

Somewhat agree

Strongly agree

10-Do you think that if his relatives were caregivers, they would support his approach? (V2)

Strongly disagree

Little agree

Neither agree nor disagree

Somewhat agree

Totally agree
