# Supplementary material for: Insights into medical students’ perceptions of work culture during the COVID-19 pandemic: a mixed method study
Source: BMC Med Educ. 2024 Jan 3;24:21. doi: 10.1186/s12909-023-04936-4 (PMC10765811; doi:10.1186/s12909-023-04936-4)
Supplement: Supplementary file 3 — Supplementary Material 3 [file 12909_2023_4936_MOESM3_ESM.docx]

Laura is a 4th-year medical student living with her diabetic and overweight father. She is very excited to start her first visceral surgery internship. As soon as her internship begins, the white plan is activated due to the health crisis linked to COVID. She quickly understands that the internship will not be able to proceed as planned. All non-urgent operations are canceled, the number of students on placement is too high in relation to the activity. She is asked to go home, to confine herself like the rest of the population, and to come only for the emergency room shifts. She does not intend to stay "doing nothing" in the middle of a health crisis. She immediately volunteers to be a backup for the health care teams.

Laura is not called in to help, although other students in her class have been. She feels helpless and useless. She feels that the others will experience something unique, that they will be united by this experience. She fears being stigmatized for her inaction. She thinks that she was turned down because of her father's vulnerability. He suggests that she move in with her mother, who is not medically vulnerable, and offer to help again.

Laura is reluctant to apply again. She has found her bearings in the confinement and enjoys the new, less hectic pace. She decides to remain confined to her father's home and not offer her services. While talking with a friend from her graduating class who was hired as a nurse, she learns that a special bonus would be offered to the caregivers. This possible bonus finally encourages her to go and live with her mother. She applies again and quickly receives an offer. She leaves for an accelerated training course in nursing techniques. At the end of this short training course, she feels that she has mastered the skills of the nursing profession and feels ready for her future duties. Just as she is about to take up her duties, a visit to the occupational medicine department detects a probable immune deficiency. This discovery is a shock because it calls into question her hiring and more generally the specialties she will be able to practice. She collapses.

1-Do you think Laura's choice to volunteer immediately is appropriate? (W1)

Strongly disagree

Agree a little

Neither agree nor disagree

Somewhat agree

Strongly agree

2-Do you think that only people who have been confronted with this type of decision can understand it? (V2)

Strongly disagree

Agree a little

Neither agree nor disagree

Somewhat agree

Strongly agree

3- Do you think Laura might have difficulties integrating with the people who were mobilized? (V2)

Strongly disagree

Little agree

Neither agree nor disagree

Somewhat agree

Strongly agree

4-Do you think Laura may be stigmatized in the future for her choice not to remodel? (W1)

Strongly disagree

Disagree a little

Neither agree nor disagree

Somewhat agree

Strongly agree

5 Do you think Laura will miss out on some of her learning because of her non-commitment (V2)?

Strongly disagree

Somewhat agree

Neither agree nor disagree

Somewhat agree

Totally agree

6- Do you think that financial motivation is likely to change the image of the work of a caregiver? (V1)

Strongly disagree

Little agree

Neither agree nor disagree

Somewhat agree

Strongly agree

7-Do you think that Laura's nursing education may change her perception of nursing work ? (T2)

Strongly disagree

Disagree a little

Neither agree nor disagree

Somewhat agree

Strongly agree

8-Do you think that the nursing education Laura received will enable her to develop distinctive skills ?(S1)

Strongly disagree

Somewhat agree

Neither agree nor disagree

Somewhat agree

Strongly agree

9-Do you think that the discovery of her disability (immune deficiency) will change Laura's feeling of belonging to the medical profession? (L2)

Strongly disagree

Little agreement

Neither agree nor disagree

Somewhat agree

Strongly agree

10- Do you think that the COVID crisis will permanently change the practice of medicine (T1) ?

Strongly disagree

Somewhat agree

Neither agree nor disagree

Somewhat agree

Strongly agree
