# Supplementary material for: Insights into medical students’ perceptions of work culture during the COVID-19 pandemic: a mixed method study
Source: BMC Med Educ. 2024 Jan 3;24:21. doi: 10.1186/s12909-023-04936-4 (PMC10765811; doi:10.1186/s12909-023-04936-4)
Supplement: Supplementary file 4 — Supplementary Material 4 [file 12909_2023_4936_MOESM4_ESM.docx]

Alex is in geriatric training, the epidemic linked to the coronavirus arrives in France, and the information coming from Italy is worrying. The emergency and resuscitation services are overwhelmed, the number of deaths is increasing in an alarming way, and the data confirm that the elderly are more at risk of death. During a staff discussion, he learns that the Italian Resuscitation Society expects to work with an imbalance between clinical needs and available ICU resources. As a result, they are preparing for decisions similar to the field of disaster medicine, which often tends to favor patients with a greater chance of therapeutic success. Specific criteria and indications are developed after much ethical reflection for this type of context, but the majority of hospital caregivers, due to their culture and training, are not used to reasoning with triage criteria. For the first time, it is explained to him that the way of working can change several times during the same day, due to clinical, logistic, or organizational problems. Alex is concerned that the same situation exists in France and that he will have to witness complex ethical choices. He cannot imagine being able to make these types of decisions if he has the responsibility. He thinks that the coronavirus crisis is more social than medical and that the situation is not comparable to an earthquake or a bombing. For him, the lack of means which is sometimes announced does not justify this type of recommendations.

1-Do you think that the health crisis linked to the coronavirus is similar to disaster medicine? (R2)

Not at all agree

Agree a little

Neither agree nor disagree

Somewhat agree

Strongly agree

2-Do you think that the coronavirus crisis is more social than medical ?(R1)

Strongly disagree

Somewhat agree

Neither agree nor disagree

Somewhat agree

Totally agree

3-Do you think Alex is too young in the business to be able to judge Italian recommendations objectively? (W2)

Strongly disagree

Disagree a little

Neither agree nor disagree

Somewhat agree

Strongly agree

4-Do you think that training in the theoretical basis of disaster medicine would change Alex's perspective? (L4,T3)

Strongly disagree

Agree a little

Neither agree nor disagree

Somewhat agree

Strongly agree

5-Do you think that only field experience allows Alex to form an objective opinion? (S1)

Strongly disagree

Somewhat agree

Neither agree nor disagree

Somewhat agree

Strongly agree

6-Do you think that following the recommendations of experts is effective in supporting the health care system? (R3)

Strongly disagree

Somewhat agree

Neither agree nor disagree

Somewhat agree

Strongly agree

7- Do you think that if Alex was faced with an ethical choice, he would have a different image of his job? (W2)

Strongly disagree

Agree a little

Neither agree nor disagree

Somewhat agree

Totally agree

8- Do you think that only people who have been confronted with this type of decision can understand it? (V2)

Strongly disagree

Little agree

Neither agree nor disagree

Somewhat agree

Strongly agree

9-Do you think that the situations Alex may encounter will allow him to develop distinctive skills? (S1)

Strongly disagree

Disagree a little

Neither agree nor disagree

Somewhat agree

Strongly agree

10-Do you think that for Alex, this potential internship experience could have an impact on his personal development? (W3)

Strongly disagree

Disagree a little

Neither agree nor disagree

Somewhat agree

Strongly agree
